# Gene Signatures Induced by Ionizing Radiation as Prognostic Tools in an In Vitro Experimental Breast Cancer Model

**DOI:** 10.3390/cancers13184571

**Published:** 2021-09-12

**Authors:** Gloria M. Calaf, Leodan A. Crispin, Debasish Roy, Francisco Aguayo, Juan P. Muñoz, Tammy C. Bleak

**Affiliations:** 1 Instituto de Alta Investigación, Universidad de Tarapacá, Arica 1000000, Chile; kcrispi@gestion.uta.cl (L.A.C.); jpmunozb@academicos.uta.cl (J.P.M.); Tammy.BLEAK@biofiredx.com (T.C.B.); 2 Center for Radiological Research, Columbia University Medical Center, New York, NY 10032, USA; 3 Department of Natural Sciences, Hostos College of the City University of New York, Bronx, NY 10451, USA; DROY@hostos.cuny.edu; 4 Laboratorio Oncovirología, Programa de Virología, Facultad de Medicina, Instituto de Ciencias Biomédicas, Universidad de Chile, Santiago 8380000, Chile; faguayo@med.uchile.cl

**Keywords:** breast cancer model, radiation, gene expression, estrogens

## Abstract

**Simple Summary:**

The present work analyzed the expression of genes involved in radiation, using an in vitro experimental breast cancer model developed by the combined treatment of low doses of high linear energy transfer (LET) radiation α particle radiation and estrogen yielding different stages in a malignantly transformed breast cancer cell model called Alpha model. Results showed important findings of genes involved in cancers of the breast, lung, and nervous system, and others. Most of those genes analyzed in these studies such as *ATM*, *selenoproteins*, *GABA* receptor, *interleukins*, *epsin*, and cathepsin inhibitors like *stefins*, and *metallothioneins* can be used for new prognostic tools and future therapies since they affect cancer progression and metastasis. In conclusion, gene signature demonstrated to be specific to cell line types, hence cell-dependency must be considered in future radiotherapy treatment planning since molecular and clinical features affect such results. Thus, using gene technology and molecular information is possible to improve therapies and reduction of side effects.

**Abstract:**

This study aimed to analyze the expression of genes involved in radiation, using an Affymetrix system with an in vitro experimental breast cancer model developed by the combined treatment of low doses of high linear energy transfer (LET) radiation α particle radiation and estrogen yielding different stages in a malignantly transformed breast cancer cell model called Alpha model. Altered expression of different molecules was detected in the non-tumorigenic Alpha3, a malignant cell line transformed only by radiation and originally derived from the parental MCF-10F human cell line; that was compared with the Alpha 5 cell line, another cell line exposed to radiation and subsequently grown in the presence 17β-estradiol. This Alpha5, a tumorigenic cell line, originated the Tumor2 cell line. It can be summarized that the Alpha 3 cell line was characterized by greater gene expression of *ATM* and *IL7R* than control, Alpha5, and Tumor2 cell lines, it presented higher *selenoprotein* gene expression than control and Tumor2; *epsin 3* gene expression was higher than control; *stefin A* gene expression was higher than Alpha5; and *metallothionein* was higher than control and Tumor2 cell line. Therefore, radiation, independently of estrogen, induced increased *ATM*, *IL7R*, *selenoprotein*, *GABA* receptor, *epsin*, *stefin*, and *metallothioneins* gene expression in comparison with the control. Results showed important findings of genes involved in cancers of the breast, lung, nervous system, and others. Most genes analyzed in these studies can be used for new prognostic tools and future therapies since they affect cancer progression and metastasis. Most of all, it was revealed that in the Alpha model, a breast cancer model developed by the authors, the cell line transformed only by radiation, independently of estrogen, was characterized by greater gene expression than other cell lines. Understanding the effect of radiotherapy in different cells will help us improve the clinical outcome of radiotherapies. Thus, gene signature has been demonstrated to be specific to tumor types, hence cell-dependency must be considered in future treatment planning. Molecular and clinical features affect the results of radiotherapy. Thus, using gene technology and molecular information is possible to improve therapies and reduction of side effects while providing new insights into breast cancer-related fields.

## 1. Introduction

According to the Health Physics Society, radiation is defined as energy that travels from a source through space as waves or particles and it can penetrate different materials. The electromagnetic spectrum consists of different wavelengths and frequencies, in which non-ionizing (low frequency) and ionizing (high frequency) radiation are found [1]. The non-ionizing radiation has the energy to move atoms in a molecule, but not enough energy to remove electrons from atoms [2]. On the other hand, ionizing radiation is part of the shortwave-type of radiation, which has sufficient energy to remove electrons by an ionization process [3]. Alpha particles, beta particles, and gamma/X-rays are the principal ionizing radiation forms produced during radioactive decay [2].

Radiation can damage cellular structures such as lipids, proteins and DNA [4]. The degree of damage will depend on the type, quantity of radiation, and energy involved and such damage will also depend on the cells themselves, since some types are more sensitive than others; thus, the ability to induce damage until cellular destruction has served as an alternative in the medical field for cancer-radiation therapy [5]. However, the safety levels and the effects this can cause during medical intervention have brought several areas of investigation; nonetheless, some well-known effects of radiotherapy are the radiation-induced effects that rely on intercellular communications, leading to more complex cell responses such as long-term radiation-induced effects and off-target effects [6]. The bystander effect is an example of off-target effect since irradiated cells can convey manifestations of damage to neighboring cells not directly irradiated as defined by the United Nations Scientific Committee on the Effects of Atomic Radiation Report, similarly, the abscopal effect consists in the radiation-response of a tissue distant from the area directly exposed to radiation [7]. Systemic and distant effects appear in the literature under the name of radiation-induced bystander effect (RIBE), involving a variety of chemical signals/molecules to propagate these off-target effects [8].

The dynamism of the signaling pathways must be considered to assess the radiation-induced effects [9], particularly, in the study of carcinogenesis. Thus, this work aims to compare the expression of genes involved in cell transformation induced by radiation, using an experimental breast cancer model developed by the combined treatment of high linear energy transfer (LET) radiation and estrogen.

## 2. Radiation Overview

Two types of radiation can be distinguished: non-ionizing and ionizing radiation; the first radiation comprises optical radiation and electromagnetic fields [10]. The optical category is divided into ultraviolet, visible, and infra-red subcategories, while the electromagnetic one can be subdivided depending on the radiofrequency [10,11]. Non-ionizing radiation can be obtained from several natural sources like the Sun and lighting, or man-made sources as those used in industrial/medical applications and wireless communications [10]. On average, a person receives approximately 2.4 mGy of natural-originated radiation every year, and this can vary according to their geographical location, for instance, countries such as Brazil, India, and China are among those with high levels of terrestrial radiation [12,13]. Ionizing radiation comprises electrically charged particles (ions), positive ones such as alpha particles and negative ones such as electrons [11]. This radiation was found after the discovery of X-rays in 1895 [14]. According to the Advisory Committee on Human Radiation Experiments alpha, beta, and gamma/X-ray radiation are the most known ionizing radiation; alpha particles are formed by two neutrons and two protons from the nucleus of the atom during the decrease of the atomic mass number and reduction of the atomic number; it results from the radioactive decay of heavy elements such as plutonium, radium, or thorium and its weight does not allow them to travel far away, being stopped by a piece of paper, and although these particles cannot pass through paper or our skin, if they are released into the body from a radioactive source, they can affect cells in our body, damaging the cells and the DNA [15]. Unlike alpha particles, beta particles are negatively charged when emitted during radioactive decay [2]. Even though these particles can reach longer distances, they can be blocked by a thin layer of substance; however, if they are swallowed or inhaled the damage can be high as that caused by alpha particles [2]. Regarding gamma rays, this radiation is frequently emitted during the radioactive decay along with alpha or beta particles, they are high-energy photons. Gamma radiation is high-energy electromagnetic radiation emitted along with alpha and beta particles during radioactive decay. Gamma particles are pure energy. Different from alpha and beta particles, gamma particles can easily penetrate the skin and cause serious tissue and DNA damage [2]. Similarly, X-rays are also pure energy but are emitted from parts of the atom different from the nucleus. This radiation is widely used in the medical field and industrial processes [2,15]. Other types of ionizing radiation can be found, such as cosmic radiation that penetrates our atmosphere and comprises mainly protons, alpha particles, and heavier atomic nuclei [16].

## 3. Radiation and Its Biological Effects

Radiation has been used in fields such as academics, agriculture, archeology, space exploration, and communications among others [17]. The use of radiation in medicine goes back to 1895 with the discovery of X-rays by Wilhelm Roentgen, who was the first person to obtain a view of the inside of the body from outside, receiving the first Nobel Prize for physics in 1901 [18]. Radiation is also important in nuclear medicine; a few irradiated radioisotopes are used for diagnostics and treatment [14]. Thus, ionizing radiation has become a good alternative for cancer therapy since it can easily get through the tissues to reach a specific area with no further surgical procedure [19]. Radiotherapy is one of the most used therapies and it has been a treatment in about 50% of total cancers, including breast cancer, as a single treatment or combined with other therapies [20,21].

One important characteristic of ionizing radiation is that it can randomly penetrate different tissues, reaching different cells, damaging them according to the dose received and not solely by the cells exposed [22]. Ionizing radiation can modify and damage DNA, RNA, and cell membrane components such as lipids and proteins by direct ionization or by water radiolysis [4]. The latter involves several reactive oxygen species (ROS), which are generated by water radiolysis, a mechanism recognized as the main contributor to cell death and tissue damage [23,24]. However, a certain degree of alterations has been proposed to the self-correction capacity of the DNA that may lead to mutations that can eventually be part of carcinogenic processes [25]. The existence of dynamic signaling pathways has made more complex the study of radiation-induced effects and their biological consequences [4].

Patients with previous cancer and who have received repeated radiotherapy have a higher probability of having their surrounding cells irradiated [14]. Different in vitro and in vivo studies have been done regarding the bystander effect, using different energy levels of radiation, different cell types and culture systems, and animal models [26,27,28,29,30,31,32]. The bystander effect was first described by Parsons in 1954 [33]. This effect consists of the genetic alteration of cells surrounding the directly radiated cells [22]. Thus, normal tissue can receive high doses of ionizing radiation when radiotherapy is applied to target organs [34]. Some have suggested that this phenomenon is due to the release of several factors such as growth factors, cytokines, and ROS into the media [35,36,37]. This communication can be possible by paracrine secretion and hemichannels formed by connexins, and by pannexins, both trans-membrane types of proteins [38,39].

RIBE is the response of non-targeted cells or tissues located near cells and tissues directly exposed to ionizing radiation [40]. In vitro and in vivo models have demonstrated RIBE in different cells [41,42,43], and these results have changed the idea that only directly exposed cells can undergo genetic alterations, such as cell death, metabolism, genomic instability, and gene expression have been reported in non-targeted cells [41,44,45,46,47,48,49,50]. RIBE can cause neoplastic transformations and changes in the cell cycle in a transmissible and long-term fashion [51]. It has been reported a wide connection with the hallmarks of cancer and RIBE; inducing angiogenesis (HIF-1, JAK-STAT/Akt), resisting cell death (autophagy, cAMP, rescue effect), inducing proliferative signaling (TGF-b, PCNA, CDC2), evading growth suppressors (CDKN1A, TP53), avoiding immune destruction (IL-6, CSF), deregulating cellular energetics (MtDNA, ROS, CYTc), inducing genomic instability (telomeres), promoting tumor inflammation (COX-2, TNFa, ROS), and inducing metastasis and invasion processes (AMPK/NFkb, JAK/STAT/Akt, VEGF/MMP2) [52]. Even though this effect has been reported by some authors, the exact mechanism and clinical relevance are still unknown [40,52].

Similarly, radiation-induced malignancies (RIMs), late side effects of radiotherapy, have been reported in adult and pediatric cancer survivors [53]. One possible explanation is the mutagenesis of the normal tissue [53]. However, the specific mechanism of RIMs is not well-defined yet, and other factors such as chemotherapy, exposure to environmental compounds, and genetic predisposition among others can contribute to carcinogenesis after radiotherapy [33].

Epidemiological studies carried out after the Nagasaki and Hiroshima atomic bomb explosions have supported the idea of radiation as a carcinogenic component and the risk of exposed people to develop diseases in the bladder, female breast, lung, brain, thyroid gland, colon, esophagus, ovary, and other organs have increased and persisted up to now [54,55,56,57]. Cancers induced by radiation appear late, even after 10 or more years and the risk can be present even after 30 years [11].

Therefore, the atomic bomb data supports one criterion described by Cahan, et al. [58] regarding the second effect produced by radiation, the RIMs, which must be originated in irradiated areas, taking at least 4 years between the irradiation and the RIM to appear, the alleged induced malignancy must be studied and the tissue from which alleged malignancy arose must have been normal [53]. The International Agency for Research on Cancer (IARC) has classified radiation exposure to external gamma or X-rays, alpha, and beta particles as carcinogenic to humans [59,60].

Over the past two decades cellular and molecular mechanism evaluations have been used to understand the effects of radiation in mammalian cells [22]. The first animal models were developed using tumor-induction studies in mice and rats right after World War II [61], then in vitro models were developed in cellular organisms in the 1970s [62]. Since radiation mainly affects the nucleus, chromosomes, and DNA [11], these cellular systems were established to study the cellular and molecular responses to DNA damage and to understand the process of cancer [22]. Ionizing radiation can cause severe DNA damage such as cross-linking, DNA strand breaks, and damage to the nucleotide bases [22]. RIMs are more frequent in presence of LET radiation which consists of alpha particles and neutrons [63]. The LET radiation (x- or gamma-rays) can cause approximately 30% of direct DNA lesions and this can increase for higher LET radiations such as alpha particles [9].

However, not only the nucleus is affected but also the cytoplasm gets affected destroying enzymatic molecules and altering mitochondrial and lysosomal membranes [11]. Changes to signaling pathways and cell environment regulation have been documented too [64,65]. Radiation can also induce inflammation associated with tumor progression [66,67]; the study of tumor cell transformation is called oncogenesis [11]. In 1982 Berenblum established the different stages of carcinogenesis as initiation, promotion, and progression [68,69] with the particularity that the transformation from a benign tumor into a malign form is determined in the last stage [11]. Thus, the entire process starts in tissues where the cellular homeostasis is disrupted, especially in those tissues with an augmented cellular activity for instance, during endometrial and mammary processes upon hormonal changes, initiation is a stage in which the reparation of DNA lesion is impossible, the cell can stay in this stage indefinitely with no effect or without being recognized by the defense system since it is not phenotypically manifested [11]. Once in the promotion stage, promoters alter the normal growth process with two phases, the reversible one first and the irreversible one later; progression is defined as a stage in which changes are noticeable and those induce cell death. Some changes are genetic, gene alterations among others; this stage is characterized by a rapid proliferation (inflammation), invasive and metastasis rhythms, thus, carcinogenic factors can be classified into exogenous and endogenous factors, among them are those which involved chemical substances, physical agents, viruses, hereditary determinism and other external causes such as lifestyle or age [11].

In vitro model systems have been extensively used to gain insights into the molecular events of cancer initiation and promotion and to identify novel prognostic/diagnostic markers for various types of cancer. All these factors can cause different biological effects leading to either cellular death or cell survival carrying different mutations [11], mutations that affect different genes with eventual abnormality in gene expression, either activating the proto-oncogenes or inactivating the tumor suppressors [70,71]. These changes in gene expression are counted as steps in carcinogenesis [72]. Alterations in gene expression can disrupt signaling pathways which can induce different aberrations and process alterations; in this condition, the expression of tumor suppressor (inhibition of cell proliferation) genes, oncogenes (migration and proliferation), checkpoint genes (to control cell cycle), and those that serve as DNA repair are affected. As a result, the cells acquire a modification in their phenotype which can be reflected in the change of gene expression, therefore, this multi-step process can trigger an initial stage of tumor growth with consequent loss of cell-cell adhesion, migration, and invasion to other tissues/organs through blood vessels [73].

An important therapeutic modality in the treatment of cancer is radiation therapy since provides curative and palliative strategies for disease control [74]. The principal target of radiation is DNA damage, and its repair is crucial to determine tumor cell death [75]. The radiation is considered a local target to control malignant lesions; however, adding systemic treatments is needed to provide radiosensitizing effects to tumors and to manage metastasis. The combination of radiation and chemotherapy has become very common for many years [76]. However, tumor control remains poor in many locally advanced cancers such as non-small-cell lung cancers, gliomas, and others that are considered radioresistant [77,78]. It has been reported that the mechanisms of radiation resistance involve inhibition of apoptosis [79], or alterations in DNA repair pathways [80], or processes inducing necroptosis, and autophagy [81,82]. Therefore, mechanisms for strategies to study radioresistant tumors are essential, but besides DNA damage, radiation also generates reactive oxygen species (ROS) which can cause the oxidation of biomolecules, such as lipid oxidation [83]. It has also been hypothesized that inefficacy of radiation can be due to the process known as ferroptosis and that inducers of such a process may be effective radiosensitizers that can expand the efficacy for radiation therapy [84]. Ferroptosis is induced when phospholipid-PUFA peroxidation overwhelms cellular defense systems, such as the capacity of the glutathione phospholipid peroxidase 4 (GPX4) and the CoQ10-regenerating enzyme FSP1 [85]. Ye, et al. evaluated the extent of these effects of radiation by measuring DNA breaks and caspase activation in HT-1080 cells cotreated with radiation and ferroptosis inducers confirming the role of ferroptosis inducers as radiosensitizers [86]. Other authors confirmed that ionizing radiation induced ferroptosis in cancer cells through ROS formation and by affecting ACSL4 expression, a lipid metabolism enzyme required for such process; such action resulted in increased lipid peroxidation and ferroptosis and they demonstrated that ACSL4 ablation abolished the ferroptosis and promoted radioresistance; ferroptosis, a form of regulated cell death caused by lipid peroxidation, has been recently identified as a natural tumor suppression mechanism even in cancer cells [87]. Other authors also demonstrated that GPX4 is a key regulator of ferroptosis and plays a crucial role in converting lipid hydroperoxides to non-toxic lipid [88,89]. Besides, glutathione (GSH) acts as an essential cofactor for GPX4. Inactivation of GPX4 or depletion of GSH accumulates lipid hydroperoxides, eventually leading to the induction of ferroptosis [88,90,91,92].

## 4. Radiation Effects and Gene Expression in Other Organs

There are some gene alterations in cells under RIBE and a previous study reported changes in the expression of proteins associated with the proliferation process, particularly cell-cycle related proteins; it was demonstrated that RIBE induced gene expression changes in a population partially exposed to radiation, where only 8% of the total population of normal human fibroblast were exposed to 0.6 cGy to 1 cGy alpha particles, and showed overexpression of p53 and p21 (Waf1) and downregulation of p34cdc2, cyclin b1, and rad51 [93].

One mechanism to explain this effect is the stress-inducible alterations which are possible through the gap junction intercellular communication under stress conditions [94,95]. Similar to those alterations obtained through our alpha model in which cellular communication is prompt to be affected due to the alteration of IL gene expression leading to inflammatory responses thus affecting the intercellular communication.

In HepG2 hepatoma cells exposed to alpha particles, CDKN1A, and TP53 genes were overexpressed in bystander cells [96]. The expression expressed by bystander cells is similar to those irradiation direct-exposed cells, being similar pathways affected [40]. For instance, in K562 cells 72% of genes changed similarly in those cells exposed to direct and non-direct irradiation [97]. Thus, the cell cycle may be affected with alpha particles in surrounding tissues due to its direct alteration demonstrated in the alpha model upon direct irradiation.

Other studies demonstrated that ionizing radiation conditioned media transferred information from the irradiated cells (such as T98G-brain of human origin) to bystander cells after gamma irradiation (0.5–1 Gy) since it significantly increased the expression of CXCR1 [98], which encodes for interleukin 8 receptor, similarly, breast cells from the model were affected by direct radiation in terms of IL; in addition, another effect related to RIBE was observed, modulation of miRNA [99,100,101,102]. Another effect related to RIBE is the epigenetic control over gene expression reported by some authors [99,103]. A study to evaluate RIBE in a more complex system was done in rats, where the liver was directly irradiated (0.125 Gy); the gene expression of the brain showed that at least 22 gene profiles were altered [104]. Results from another study indicated that NF-kB was fundamental in the RIBE phenomenon [105].

RIBE is one example of the long-term effects of radiation and the capacity to induce important changes not only in the direct irradiated cell/organ but also in their surroundings, leading to a pool of interesting areas of investigation; thus, the comparison with an in vitro model already transformed by irradiation can contribute to understanding the induced effects, evaluate, and estimate the potential evolution based on the simple comparison of these systems [105].

Some genes have shown certain grade of alteration in those direct radiation-induced cancer, for instance, RAS family in mouse lymphomas, C-MYC in murine osteosarcomas or MDM2 in X-ray transformed foci [22,106], and loss of G1 check point in human tumor cells and mouse 10T1/2 cells [107,108,109]. Similarly, in the Alpha model, the *ATM* gene was an important check point affected by direct radiation leading to the idea that radiation can influence different cells causing cellular transformation by different signaling pathways [106].

Another direct type of radiation is hadrontherapy, which uses charged beams with particles as carbon ions [110]. Different from RIBE, this radiation is more localized and precise, thus offering more efficient DNA damage and death in tumor cells [111,112,113]. It has been approved as radiotherapy for different cancers [114,115]. This type of therapy has been also evaluated regarding the effects on gene expression induced in different cell types, particularly, in the prostate adenocarcinoma cell line (PC3) where it was found that several genes were associated with cell cycle and cell motility being down-regulated (after 2.0 Gy carbon ion radiation), such as NEXN, CCDC88A, FN1, MYH9, MYH10, and ROCK1 important in the Rho-pathway associated with migration; in addition, this behavior responded to a dose-dependent relationship [110].

Other studies have been done in human immortalized breast cells such as MCF-10A, MCF7, and MDA-MB-231 and normal primary breast cells as HMEC, BCpc7, and BCpcEMT to evaluate the effects of the intraoperative electron radiotherapy on gene expression exposed at 9 and 23 Gy for 24 h [20], typical for this type of therapy [20,116,117]. It was observed in this study that in those primary cells the genes associated with the cell cycle were CDCA5, CDC6, and CDCA7; some transcription factors as E2F1, E2F2, and others as MCM10 and MCM6 were also observed, similarly, in those breast tumor cells, alterations were found in cyclins, minichromosome maintenance (MCM), proto-oncogene (MDM2), and others involved in cell progression (cyclin-dependent kinase, CDK). In addition, the same study concluded that different cellular types responded in different ways upon IR [20]. There was a hereditary factor in which a genetic instability was established along with generations [118], augmenting the rate at which these transformations would appear spontaneously, and this mutation rate could be present for about 30 generations post-irradiation [22]. In line with this, the analysis of gene alteration upon massive radiation exposure has also contributed to understanding the effects of radiation on human genes.

After the nuclear reactor accident occurred in Chernobyl in 1986 different epidemiological studies were carried out to evaluate the impact of this extended irradiation exposure on the population [119]. It was proposed that after radiation exposure at a young age cancers such as papillary thyroid cancer would appear [120,121]. Likewise, an increase in the incidence of thyroid carcinoma in children was observed in Belarus and Ukraine [122]. The Thyroid Cancer Committee suggested a national registry only for thyroid cancer in young people to have a better perspective of the epidemiological tendency [123]. Thus, RNA samples were taken from papillary tumor tissue from post-Chernobyl patients who were diagnosed up to 15 years after the exposure; the irradiation dose was estimated to be approximately 0.15–1.0 Gy for 90%, 1–5 Gy for 9%, and more than 1 Gy for about 1% of the evacuated kids [124]. After the genome microarray screening, gene expression was quantified of growth factors such as VEGF-A, EGFL9, PDGFC and PDGFRB, IGF1R, and IGBP1 that were found to be increased in the post-Chernobyl group compared to the control group; such results are being aligned with other authors that associated lymph node metastases and aggressive behavior with increased serum concentrations of VEGF-A and EGFR protein in patients older than 45 years old [125,126,127,128]. In addition, another gene such as the MMP1 linked with invasive processes was found augmented in those patients irradiated in the Chernobyl accident [119,129].

As it was mentioned before, RIMs have been considered under the umbrella of genetic modifications in cancer and non-genetic alterations have also been reported such as epigenetic changes, phosphorylation, methylation, and acetylation, and histone modification under radiation [130,131,132,133], affecting the DNA repair and intracellular processes such as cell cycle [25]. In a recent study, human skin primary cells were chronically irradiated with ionizing irradiation at different dose rates (6, 8, 12 and 20 mGy/h). Results indicated that low doses of irradiation hampered cell proliferation in a dose-dependent fashion, a decrease in some proteins related to DNA replication and cell cycle progression that comprised MCM 2-7 and SMC1A/3, and also an increase of (p16) CDKN2A and (p21) CDKN1A [25], which were associated with senescent cells while other genes such as EGFR1, IL6, MMP family, SERPINE1 and CCNA2 were upregulated after chronic irradiation of human skin cells [134,135,136]. Therefore, important changes in genes associated with the cell cycle process, inflammation and homeostasis, and detoxification were shown; changes eventually pivotal in processes that involve metastasis and proliferation.

## 5. Alpha Model, Radiation, and Carcinogenesis

Since the effects of continuous exposure to the ionizing radiation on non-tumorigenic breast cells have not been completely uncovered yet, an experimental breast cancer model to explore this area was developed by Calaf and Hei [137].

The human breast epithelial cell line MCF-10F, spontaneously immortalized and derived from the breast tissue of a 36-year-old female, has the morphological characteristics of normal breast epithelial cells [138]. The MCF-10F cell line has been used to detect sensitivity to both chemical carcinogens such as 7,12-dimethylbenz(a)anthracene (DMBA) and benzo(a)pyrene (BP) [138] and environmental carcinogens such as ionizing radiation [139].

**Figure 1:**
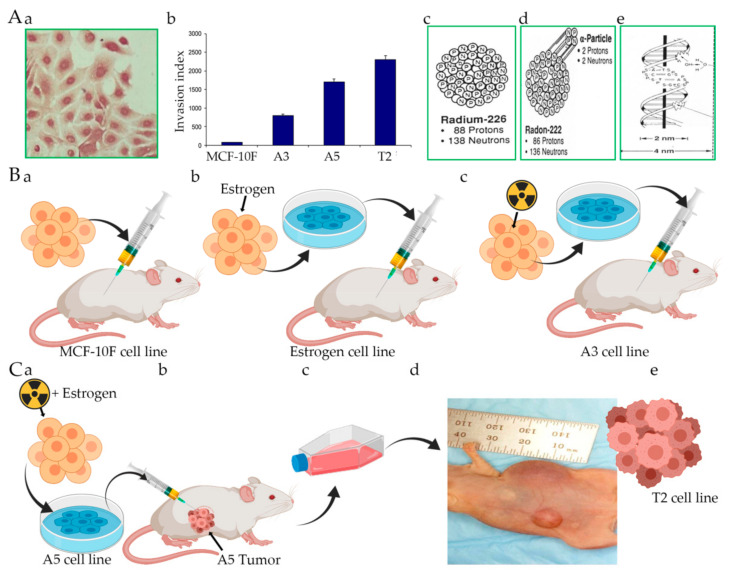
The model consists of human breast epithelial cells in different stages of transformation: (i) parental cell line MCF-10F; (ii) an Estrogen cell line (MCF-l0F continuously grown with estradiol at 10^−8^ (Estrogen); (iii) a malignant and non-tumorigenic cell line (60/60 cGy, named Alpha3), non-malignant cell line (Alpha3); and (iv) a malignant and tumorigenic cell line (60/60 cGy plus estrogen, named Alpha5) and the Tumor2 cell line derived from the nude mouse xenograft of the Alpha5 cell line injected into nude mice. This Figure shows (**A**) (**a**) MCF-10F cell line grown in monolayer; (**b**) Invasion Index with MCF-10F, Alpha3 (A3), Alpha5 (A5) and Tumor2 (T2) cell lines; (**c**) Radium 226, (**d**) Radon 222 and (**e**) scheme of DNA; (**B**) Mouse injected with (**a**) the control, (**b**) Estrogen, (**c**) A3. (**C**) (**a**) A5 cell line (radiation + estrogen); (**b**) Mouse injected with A5 cell line, (**c**) T75 flask with cultured A5 cell line; (**d**) Athymic mouse that shows mammary tumor, giving rise to (**e**) T2 cell line.

An in vitro experimental breast cancer model (Alpha-model) developed by exposure of the immortalized human breast epithelial cell line, MCF-10F, to low doses of high LET α particle radiation (150 keV/μm) and subsequent growth in the presence or absence of 17β-estradiol was used in this study (Figure 1). These studies indicated that the combined treatment of ionizing radiation and estrogen yielded different stages in a malignantly transformed breast cancer cell model system, which was called, the Alpha model system [137]. This model consisted of human breast epithelial cells at different stages of transformation: (i) a control cell line MCF-10F (C); (ii) MCF-l0F continually treated with estradiol at 10^−8^ M named estrogen cell line (E); (iii) a non-malignant cell line, Alpha3 (A3) cell line; and (iv) a malignant and tumorigenic cell line named Alpha5 (A5) cell line and the Tumor2 cell line derived from cells originating from a tumor after injection of A5 cells in nude mice, named after T2 cell line. Using this model system, altered expression of different cell communication molecules was detected in the parental, non-tumorigenic, and malignantly transformed cell lines originally derived from the parental MCF-10F human cell line. Previous work demonstrated that estrogen was a prerequisite for the process of high LET radiation-induced carcinogenesis [137,140]. Several phenotypic properties such as growth rate, anchorage-independent growth, and invasive characteristics have also been reported to be grossly similar during the transformation process induced by chemical carcinogens [138,141,142,143,144,145,146] and environmental factors, e.g., ionizing radiation [139]. The chemo-invasion or the ability of transformed cells to infiltrate the basement membrane in vitro was correlated well with the in vivo malignant characteristics.

This study revealed that in the Alpha model, a breast cancer model developed by the author, the cell line transformed only by radiation, independently of estrogen, was characterized by greater gene expression than other cell lines. Understanding the effect of radiotherapy in different cells will help us improve the clinical outcome of radiotherapies. The work aimed to identify the gene signature that could be demonstrated to be specific to tumor types. Cell-dependency must be considered in future treatment planning and the molecular and clinical features are important for radiotherapy. Thus, using gene technology and molecular information is possible to improve therapies and reduction of side effects. Therefore, these findings will provide new insight into breast cancer-related fields.

## 6. Gene Expression Induced by Radiation

**Figure 2:**
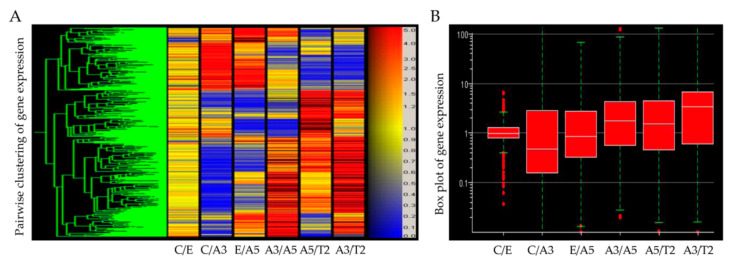
Heatmap of Affymetrix array (U133A) data that allows to compare the following cell lines: MCF-10F (C) /Estrogen (E); C/Alpha3 (A3); E/Alpha5 (A5); A3/Alpha5; A5/Tumor2 (T2) and A3/T2 [153]. The red color indicates a higher expression in the first cell line, blue a lower expression, and yellow equal expression in both cell lines (**A**). The box plot of the gene summarizes the range of differential gene expression in the same pairwise cell line comparisons (**B**).

**Figure 3:**
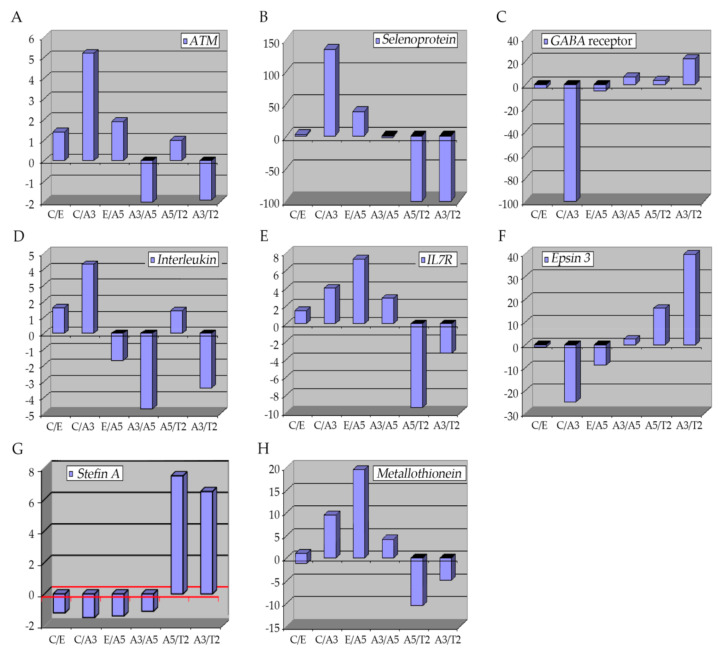
Graphs show the profiling of differentially expressed genes obtained through an Affymetrix array U133A data comparing these genes: (**A**) *ATM*, (**B**) *selenoproteins*, (**C**) *GABA* receptor, (**D**,**E**) *interleukins*, (**F**) *Epsin 3*, (**G**) *Stefin A (CSTA)*, and (**H**) *Metallothioneins* in Heatmap of Affymetrix array (U133A) data that allows comparing the following cell lines: MCF-10F (C)/Estrogen (E); C/Alpha3 (A3); E/Alpha5 (A5); A3/Alpha5; A5/Tumor2 (T2) and A3/T2. All graphs were obtained from a Cluster-dendrogram repository of gene expression from our laboratory for this review.

It is considered that different signaling pathways are activated in a cell under radiation directing to proliferative or cell death processes [147]. Despite the study of in vitro and in vivo systems, there is no data available regarding specific tumor suppressor inhibition or oncogene activation by radiation [22]. However, there are features of surviving irradiated cells after traditional radiation such as invasion, migration, and angiogenesis that demonstrate enhanced aggressiveness of these cells [148,149,150,151,152]. Molecular aspects induced by radiation and estrogen were analyzed in cells derived from this model to analyze the gene expression involved in several cellular processes, using an Affymetrix system (Figure 2) and the results show changes in the expression of genes as stated in Figure 3.

### 6.1. The Ataxia-Telangiectasia Mutated Gene

The ataxia-telangiectasia mutated gene (*ATM*) encodes for a 350 kDa protein serine/threonine kinase, key in the DNA-damage response elements since it can detect double-strand breaks (DSBs), fundamental in the cell-cycle checkpoint controlling [154,155,156,157]. The *ATM* gene has 66 exons with approximately 150 kb of genomic expansion [155]. Mutations of the *ATM* gene can explain ataxia telangiectasia (AT), a rare neurodegenerative disease, which is manifested clinically by skin and ocular telangiectasia, immunological deficiency, neuronal deficiency, sino-pulmonary infections, cellular sensitivity to ionizing radiation, and predisposition to cancer [158,159,160,161,162,163,164]. However, it has been estimated that approximately 2% of the adult population presents the heterozygosity for an *ATM* variant [165,166,167]. Even though this group exhibits no phenotypical abnormalities, it has been reported that *ATM* heterozygotes have a high risk of developing breast cancer, with about a 5-fold increase compared with the general population [160,168,169,170,171,172,173]. Normally, *ATM* expression is down-regulated in breast cancer tissues [173]. Locally advanced breast tumors have shown a reduced expression of *ATM* by epigenetic silencing [174].

As it was mentioned before, *ATM* mutations can increase the sensitivity for ionizing radiation, thus affecting radiation therapy producing important radio-necrosis in some cases [175].

Studies done with our in vitro experimental breast cancer model indicated that the cell line named A3 and characterized by cells transformed only by radiation had higher *ATM* expression than C, A5, and T2. Thus, the *ATM* gene has been fundamental in breast cancer progression corroborating the work of other authors [173].

### 6.2. Selenoproteins (SEPP1)

These are metalloproteins with certain characteristics that allow them to have a high affinity to metal; they present a specific amino acid, seleno-cysteine (Se-Cys) [21]. There are 25 types of selenoproteins in humans with this characteristic [176,177]. Selenoproteins are involved in several cellular processes related to metastasis, comprising cell adhesion, matrix degradation, migration, invasion, and proliferation [178]. Among these processes, roles in redox balance and calcium equilibrium have been described [178], but the metabolism of these proteins can also modify signaling pathways in cancer cells [179].

Glutathione peroxidases (GPXs), are selenoproteins that are responsible for the protection of tissues against reductions derived from the action of ROS such as those produced during ionizing radiation [180]. These enzymes are in charge of the hydroperoxide (H_2_O_2_) reduction, a type of ROS produced in the cell [181,182], thus decreasing the negative effects of ROS and contributing to the anti-metastatic function [178,183,184,185]. They are also involved in roles such as DNA-repairing and cytokine control, thus, supplements with Se have been used in chemotherapy since it was reported that it increased the selenoprotein expression in plasma [186]. Similarly, other selenoproteins that participate in redox processes during tumor progression are the thioredoxin reductases (TXNRDs) with three subtypes placed in the cytosol and nucleus, mitochondria, and sperm [187]. This selenoprotein subfamily is up-regulated in several cancers such as lung cancer [188,189], breast cancer [190,191], and astrocytomas [192].

Regarding breast cancer, it has been correlated with pro-invasiveness characteristics of the MDA-MB-231 breast cancer cell line and poor prognosis in patients with breast cancer [193]. Thus, this selenoprotein has been suggested as a target for anti-cancer therapies especially in EMT processes to reduce metastasis development [194]. Another type of selenoprotein associated with carcinogenesis and metastasis in breast cancer is the SELENOH, which has been associated with the P53 signaling pathway, key in developing cancer [195]. Studies in the breast cancer model indicated that A3 characterized by cells transformed by radiation had greater selenoprotein gene expression than C and T2.

### 6.3. GABA Receptor

γ-Aminobutyric acid (*GABA*) is the principal inhibitor in the central nervous system and it is present in the peripheral nervous system as well [196,197]. The expression of *GABA* and *GABA* receptors (*GABARAP*) is mainly found in brain structures; however, its expression can be detected in other organs like the pancreas, kidney, intestine, prostate, testis, ovaries, and liver where it can trigger hormone and neuronal activity [196]. Ionizing radiation augments the *GABA* receptors mRNA in C17.2 mouse neural stem-like cell lines and in mouse primary neural stem cells, presumably by altering the neuronal function [34]. Nevertheless, other studies have shown contrary effects that may be because of the differences in doses and time of exposure [34,198,199]. It was reported that the *GABA* signaling pathway was altered in some cancers such as pancreatic, gastric, and breast cancers, an increment in the expression of *GABA* and *GABA* receptors was found [200,201,202]. It was observed that activation of these receptors could decrease cell proliferation and migration [203,204,205]. Thus, the GABARAP system was suggested to play a role in tumorigenesis as a cell migration and proliferation inhibitor [206].

Regarding breast cancer, data suggest that *GABA* is involved in the development of this type of cancer, where low concentration is linked with a low survival rate in breast cancer patients, this may be caused by overactive GABA-T transaminase, that responds in environments rich in oxygen, typical in processes such as neo-angiogenesis during tumor progression, thus, *GABA* has been proposed as a new prognostic marker [196]. Studies in the breast cancer model indicated that A3 cell line, characterized by cells transformed by radiation, had higher *GABA* receptor gene expression than C and T2 cell lines.

### 6.4. Interleukins

Interleukins (IL) are proteins secreted primarily by CD3+ and CD4+. They belong to the cytokine superfamily with about 38 different types of IL and are responsible for the interactions between cells [207]. In tumors, cytokines collaborate with different elements such as cancer stem cells, microRNA, epithelial-mesenchymal transition (EMT) markers, and transcription factors, thus these biomolecules are involved in different processes, principally in systemic inflammation and immune system modulation, involving cell migration, proliferation, maturation, and adhesion necessary for the inflammatory response [208,209].

*IL7* is about 25 kDa and the specific gene is located in the locus 8q12–13, this interleukin can interact with the surface receptor interleukin-7 receptor (*IL7R*); which contains the *IL7R* alpha chain; errors in the expression of *IL7* and its receptor are linked to breast cancer [210] and this can promote survival and cancer cell growth in vitro and poor prognosis in humans [207].

On the other hand, radiotherapy has been reported to influence the inflammatory response by modulating the cytokines in a time- and dose-dependent manner, and such effect can last even years [211,212,213]. The mechanism proposed is that under ionizing radiation the innate immune response is induced mainly in macrophages, resulting in chronic inflammation of tissue damage and fibrosis [214]. Immunogenic cell death or danger-associated molecular patterns (DAMPs) are exerted. Eventually, such DAMPs can induce several responses in dendritic cells, myeloid-derived suppressor cells, and the release of cytokines [215]. Considering the Alpha-model, it is possible to mention that the A3 cell line, characterized by cells transformed by radiation, had higher *IL7R* gene expression than C, A5, and T2 cell lines.

### 6.5. Epsins 3

Epsins are adaptor proteins, part of a family of ubiquitin-binding endocytic proteins [216]. In mammals, three genes encode for each isoform; *epsin1*, *epsin2*, and *epsin3* [217,218,219]. These proteins have a well conserved NH_2_-terminal homology domain (ENTH), important in ubiquitination and the ENTH portion comprises about 150 amino acids and it is essential for binding inositol phospholipids and proteins [220]. Thus, involved in signaling pathways like Notch, Rho GTPase, and VEGFRs [221,222,223,224]. *Epsin* type 3 is predominately expressed in the stomach and the epithelia while types 1 and 2 are expressed in different cell types, with no specific location and repetitious functions [217,218,219]. Besides, it has been shown that these proteins are up-regulated in different cancers [225,226]. In breast cancer, *Epsin* proteins induce NF-kB, which is fundamental in developing the disease, high levels of *epsins* are associated with low relapse-free survival rates, especially in ER-negative breast cancer types [216]. *Epsin3* acts as an oncogene in ER-positive breast and other cancers such as non-small cell lung cancer [227]. Similarly, the *epsin3* protein has been identified in glioblastoma cell lines and samples of patients with glioma, and its overexpression has been shown to induce cell migration and invasion through transcription factors such as Slug, Twist, and ZEB1 promoting EMT in these glioma-type cells [227]. Studies in the breast cancer model indicated that A3, characterized by cells transformed by radiation only, had higher *epsin* gene expression than C.

### 6.6. Stefin A (Cystatin A)

To understand the role of *stefin* genes (*cystatin A, CSTA*), it is important to know that cathepsins are lysosomal proteases and they can be classified into serine, cysteine, and aspartyl cathepsins [228,229], with 11 well-identified types: B, H, L, S, C, K, O, F, V, W, and X/Z [230]. These proteases are essential in protein degradation processes, associated with phagocytosis, endocytosis, and autophagy [231], in addition to apoptosis, immune response, development, differentiation, and pro-tumorigenic functions [228,230,232]. For instance, cathepsin S type is involved in tumor progression [233], angiogenesis, tumor growth [234,235], similarly, cathepsin L is involved in neovascularization [236], migration, and invasion processes as well [237,238]. Alteration and changes in expression of cathepsins are associated with pathological circumstances, for example, they are secreted into the extracellular medium in cancer [239,240,241].

These proteases can be balanced by small naturally occurring molecules, the so-called cathepsin inhibitors [242]. These inhibitor groups comprise proteases such as cystatins, thyropins, α2-macroglobulin, cytotoxic antigen 2β, and other parts of the serpin family [243]. The inhibitors can be classified into families; family I (*stefins*), family II (cystatins), family III (kininogens), and family IV (fetuins). Particularly, *stefins A, B*, and *C* function as endogenous cysteine-cathepsins inhibitors [244], with eventual effects in tumor growth, invasion, and metastasis [245]. Besides, *stefins* A and B have demonstrated different functions in human cancers, for instance, *stefin* A has shown cancer development induction and aggressiveness at low expression levels in breast, lung, prostate, and esophageal tumors [246,247,248,249]. On the contrary, the same type of *stefin* has also demonstrated a positive clinical outcome in breast cancer when low expressed [250]. *Stefin* A has been widely observed in myoepithelial cells in breast tissue [251]. Besides, its role as a tumor suppressor in myoepithelial cells surrounding ductal carcinoma in situ samples from patients has been confirmed [251]. Studies in the breast cancer model indicated that the A3 cell line had greater *stefin A* gene expression than the A5 cell line.

### 6.7. Metallothioneins

Metallothioneins (MTs) are small (approximately 6–7 kDa) cytosolic proteins with a high content of cysteine groups (30%) [252,253]. There are four main MT isoforms in humans—MT1, MT2, MT3, and MT4—encoded by a gene located at the 16q13 locus [254]. There is evidence that connects MTs with tumor formation, progression, and drug resistance [254]. Their principal role is in homeostasis and the detoxification of heavy metals, oxidative stress, and DNA damage processes [254,255]. They bind to heavy metals (with great affinity) via the thiol binding part of the cysteine-enriched portion [254]. When MTs bind to metals such as zinc and copper, they can regulate different important processes such as cell growth, proliferation, differentiation, metastasis, and protection against oxidative radicals produced by drugs and radiation [254,256,257,258]. However, they also can bind to other metals like cadmium, mercury, and platinum, among others, to protect cells from these heavy metals [259]. The expression of MTs depends on the type of tumor suggesting a specific role in carcinogenesis [252,260,261,262]. MTs have been reported to be up-regulated in breast, ovarian urinary bladder, and nasopharyngeal cancer, and melanoma [263,264,265,266,267]. A positive correlation has been established between MTs and Ki-67, a marker of cellular proliferation in breast cancer [263,268,269]. Specific MTs such as MT1F and MT2A have been found in greater numbers in cancer in stage 3 than in stage 1 and 2 when histological samples of breast cancer were analyzed [269,270]. In addition, zinc was demonstrated to increase the expression of the vascular epithelial growth factor (VEGF) in three breast cell lines [271].

The metastatic process is also modulated by the high expression of MTs principally in lymph node metastasis in breast and other cancers [272,273]. Clinical studies have also correlated high expression of MTs with breast cancer, specially MT2A, which is overexpressed and modulates invasion and migration via MMP-9 activating signaling pathways such as AP-1 and NF-kB [274]. Likewise, MT3 increase is associated with breast cancer invasion due to regulating MMP-3 [275]. However, low expression of MT3 has been found in patients with ductal breast cancer with lymph node metastasis [276]. MT overexpression has been proposed as a tumor progression marker in breast cancer with poor prognosis (MT3) and other cancers such as ovarian, bladder, and lung cancer [265,277,278,279,280,281]. Thus, MTs modulate different processes such as tumor migration, invasion, and metastasis [282,283,284]. Regarding MTs and tumor progression, such proteins achieve their maximum level in G1/S cell cycle transition, sustaining their role in cell proliferation [285]. The *MT1H* has low expression and serves as a tumor suppressor in prostate cancer [254]. Studies in the breast cancer model indicated that the A3 cell line, characterized by cells transformed by radiation, had higher *MT1H* gene expression than C and T2 cell line.

## 7. Relationship between Genes and Clinical Aspects

Tumor progression and immunotherapy efficacy are highly influenced by the composition and abundance of immune cells in the tumor microenvironment, due to the constraints of direct measurement methods, computational algorithms are generally used to infer the features of immune cells from a large number of tumor transcriptome profiles. The TIMER2.0 webpage provides an estimate of immune infiltration levels for The Cancer Genome Atlas (TCGA) through four modules to study the relationship between immune infiltrates and genetic or clinical features, and four modules to explore cancer-related associations in the TCGA cohorts [286]. Hence, the genes studied herein were evaluated by TIMER2.0 to show whether such a gene had therapeutic target potential, to predict survival and therapy response, to discover the co-expression pattern of genes across TCGA cancer types such as breast invasive carcinoma (BRCA) and to identify the relationship between tumor gene expression and immune infiltration.

### 7.1. Genes Related to Clinical Relevance in Breast Cancer Patients

The TIMER gene outcome module provides the clinical relevance of gene expression across breast cancer. Analysis through the web showed that ATM and SEPP1 induced significantly (*p* < 0.05) increased risk in LumA BRCA patients. Those patients had significantly (*p* < 0.001) higher increased risk in Stage 4 than in other stages. However, GABARAP induced significantly (*p* < 0.05) decreased risk in all BRCA patients analyzed. Those patients had significantly (*p* < 0.001) higher increased risk in stages 3 and 4 than in other stages. Such results also indicated that IL7R had significantly (*p* < 0.05) higher expression and decreased risk in all BRCA patients. However, patients in stage 4 had significantly (*p* < 0.05) higher increased risk than in other stages. On the other hand, the analysis showed that EPN3 did not present a significant risk in tumor breast tissues derived from basal, Her2, LumA (LumA), and LumB (LumB) cell types. However, results indicated that MTR had significantly (*p* < 0.05) higher expression and increased significantly (*p* < 0.05) risk in LumA tissues and significantly (*p* < 0.05) decreased risk in basal tissues of BRCA patients. However, patients in stage 4 and LumA type had significantly (*p* < 0.05) higher increased risk than in others. Those patients with tumor tissues positive for MTR and derived from basal type did not have significant risk.

**Figure 4:**
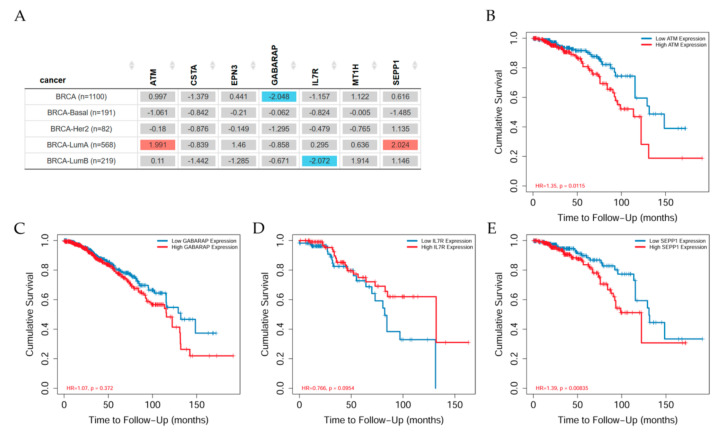
(**A**) Gene expression across various BRCA patient types. KM curves of the genes where the cumulative survival differences in low and high gene expression can be appreciated. The KM curves correspond to the cumulative survival and time to follow up (months) of (**B**) ATM, (**C**) GABARAP, (**D**) IL7R, (**E**) SEPP1 at low and high expression given by cumulative survival and time to follow-up.

Here for the gene survival a Cox proportional hazard model was used to evaluate the outcome significance of gene expression. The heatmap table shows the normalized coefficients of the genes in the Cox model. The gene outcome module was used to explore the increase risk of survival in low and high ATM gene expression across various types of BRCA patients as seen in Figure 4A–E. The heatmap show the normalized coefficient of the gene in Cox model as seen in Figure 4A. Results indicated that ATM (Figure 4B) and SEPP1 (Figure 4E) genes induced significant (*p* < 0.05) increased risk in BRCA-LumA patients than other type of breast cancer patients while GABARAP (Figure 4C) gene induced significant (*p* < 0.05) decreased risk in all BRCA patients, but IL7R (Figure 4D) in only BRCA-LumB patients. On the other hand, there was a non-significant difference in CSTA, EPN3 and MT1H.

According to results related to gene risk in the web based on Z-score, studies indicated that: ATM had increased risk (*p* < 0.05) in BRCA-LumA patients; a non-significant (*p* < 0.05) difference in risk with CSTA in all BRCA, BRCA-basal, BRCA-Her2, BRCA-LumA, BRCA-LumB patients; a non-significant (*p* < 0.05) risk with CSTA and EPN3 in all BRCA, BRCA-basal, BRCA-Her2, BRCA-LumA, BRCA-LumB patients; GABARAP had decreased risk (*p* < 0.05) in BRCA-basal, BRCA-Her2, BRCA-LumA, BRCA-LumB patients; non-significant (*p* < 0.05) difference in risk MT1H in all BRCA, BRCA-basal, BRCA-Her2, BRCA-LumA, BRCA-LumB patients; and finally, an increased risk (*p* < 0.05) in BRCA-LumA patients. Studies on gene survival from the web on breast cancer patients show the gene outcome module used to that explore the clinical relevance of low and high ATM gene expression across various types of BRCA. Gene survival used the Cox proportional hazard model to evaluate the outcome significance of gene expression. The KM curves of ATM at low and high expression is given by cumulative survival and tie to follow-up (months). Results indicated that ATM and SEPP1 genes induced significant (*p* < 0.05) increased risk in BRCA-LumA patients than other type of breast cancer patients while GABARAP gene induced significant (*p* < 0.05) decreased risk in all BRCA patients, but IL7R in only BRCA-LumB patients. On the other hand, there was non-significant difference in CSTA, EPN3 and MT1H.

### 7.2. Gene Correlation between ATM and Other Genes

**Figure 5:**
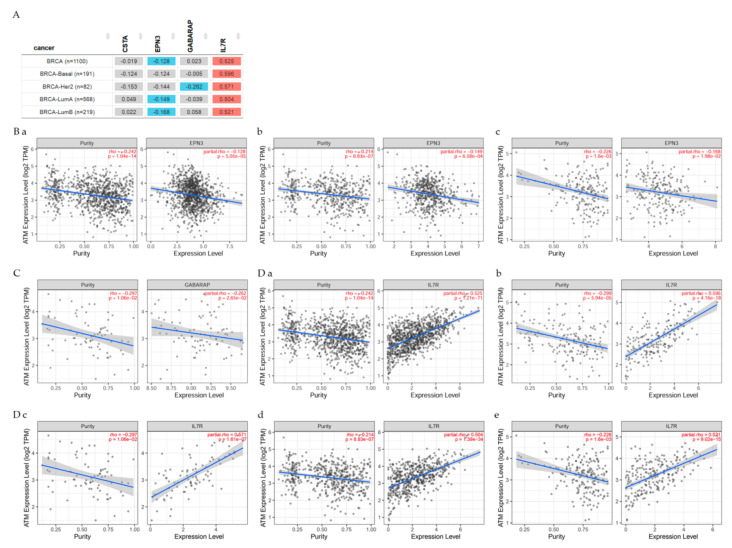
The gene correlation module used to explore the correlation between ATM and genes related to the present studies. (**A**) The table explores the correlations between ATM gene expression and other genes in BRCA patient types. These figures show the correlation between ATM gene expression and (**B**) EPN3 in (**a**) All BRCA, (**b**) BRCA-LumA, and (**c**) BRCA-LumB; (**C**) GABARAP in BRCA-Her2; (**D**) IL7R in (**a**) All BRCA, (**b**) BRCA-Basal, (**c**) BRCA-Her2, (**d**) BRCA-LumA, and (**e**) BRCA-LumB.

**Figure 6:**
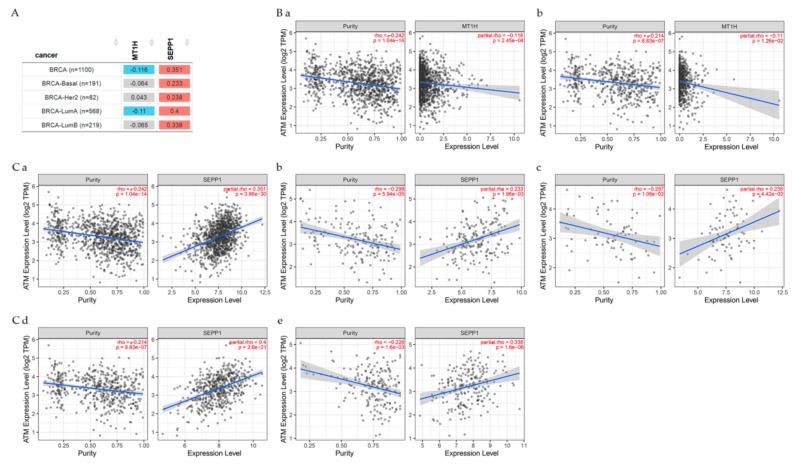
(**A**) The table explores the correlations between ATM gene expression and other genes in BRCA patient types. These figures show the correlation between ATM gene expression and (**B**) MT1H in (**a**) All BRCA and (**b**) BRCA-LumA; (**C**) SEPP1 in (**a**) All BRCA, (**b**) BRCA-Basal, (**c**) BRCA-Her2, (**d**) BRCA-LumA, and (**e**) BRCA-LumB.

TIMER2.0 analysis gene correlation module was used to explore the correlation between *ATM* and genes related to the present studies in breast cancer patients as seen in Figure 5 and Figure 6.

According to Spearman’s *p* values results indicated that ATM had: non-significant (*p* < 0.05) difference with CSTA in all BRCA, BRCA-basal, BRCA-Her2, BRCA-LumA, BRCA-LumB patients; a significant (*p* < 0.05) negative correlation with EPN3 in all BRCA, BRCA-LumA and BRCA-LumB; a significant (*p* < 0.05) negative correlation with GABARAP in BRCA-Her2; a significant (*p* < 0.05) positive correlation with IL7R in all BRCA, BRCA-basal, BRCA-Her2, BRCA-LumA and BRCA-LumB patients.

A significant (*p* < 0.05) negative correlation is seen with MT1H in all BRCA and BRCA-LumA patients; a significant (*p* < 0.05) positive correlation with SEPP1 in all BRCA, BRCA-basal, BRCA-Her2, BRCA-LumA and BRCA-LumB patients. Genes in gray color were not significantly different (*p* < 0.05) from ATM.

### 7.3. Differential Gene Expression Levels between Breast Tumors and Adjacent Normal Tissues across All TCGA Tumors

**Figure 7:**
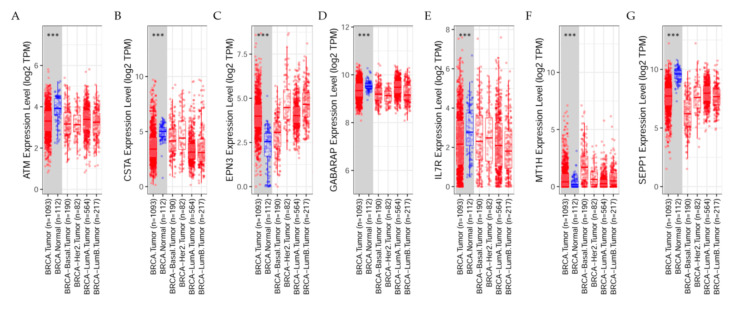
The differential gene expression levels between tumors and adjacent normal tissues. (**A**) ATM gene expression in breast tissues derived from basal, Her2, LumA, and LumB breast tissues; (**B**) CSTA, (**C**) EPN3, (**D**) GABARAP, (**E**) IL7R, (**F**) MT1H, and (**G**) SEPP1 in tumor tissues derived from basal, Her2, LumA, and LumB breast. ***: *p*-value < 0.001.

Studies from the web on breast cancer patients show the distribution of gene expression levels displayed in box plots. The statistical significance computed by the Wilcoxon test is annotated by the number of stars (*: *p*-value < 0.05; **: *p*-value < 0.01; ***: *p*-value < 0.001). Through this system was possible to identify genes that were up-regulated or down-regulated in the tumors compared to normal tissues for each cancer type as seen in Figure 7A–G.

The differential expression between tumor and adjacent tissues expression levels is displayed in the box plot. Results indicated that for ATM (Figure 7A) such expression is significantly (*p* < 0.001) higher in normal tissues than tumors, confirming its tumor suppressor gene function. The tumor tissues derived from basal, Her2, LumA, and LumB breast tissues were not significantly different between normal and tumor ones; for CSTA (Figure 7B): the expression levels are displayed in the box plot. Results indicated that such expression is significantly (*p* < 0.001) higher in normal tissues than in tumors. The tumor tissues derived from basal, Her2, LumA, and LumB breast tissues were not significantly different between normal and tumor ones; for EPN3 (Figure 7C): the expression level is displayed in the box plot. Results indicated that such expression is significantly (*p* < 0.001) higher in tumors than in normal tissues. The tumor tissues derived from basal, Her2, LumA, and LumB breast tissues were not significantly different between normal and tumor ones; for GABARAP (Figure 7D) the expression levels are displayed in the box plot. Results indicated that such expression is significantly (*p* < 0.001) higher in normal tissues than in tumors. The tumor tissues derived from basal, Her2, LumA, and LumB breast tissues were not significantly different between normal and tumor ones; for IL7R: (Figure 7E) the expression levels are displayed in the box plot. Results indicated that such expression is significantly (*p* < 0.001) higher in tumors than in normal tissues. The tumor tissues derived from basal, Her2, LumA, and LumB breast tissues were not significantly different between normal and tumor ones; for MT1H (Figure 7F) the expression levels are displayed in the box plot. Results indicated that such expression is significantly (*p* < 0.001) higher in normal tissues than in tumors. The tumor tissues derived from basal, Her2, Luml A, and LumB breast tissues were not significantly different between normal and tumor ones; for SEPP1: (Figure 7G) the expression levels are displayed in the box plot. Results indicated that such expression is significantly (*p* < 0.001) higher in normal tissues than in tumors. The tumor tissues derived from basal, Her2, LumA, and LumB breast tissues were not significantly different between normal and tumor ones.

### 7.4. Correlation of ATM Gene and Its Expression with Immune Infiltration Level in Diverse Breast Cancer Types

**Figure 8:**
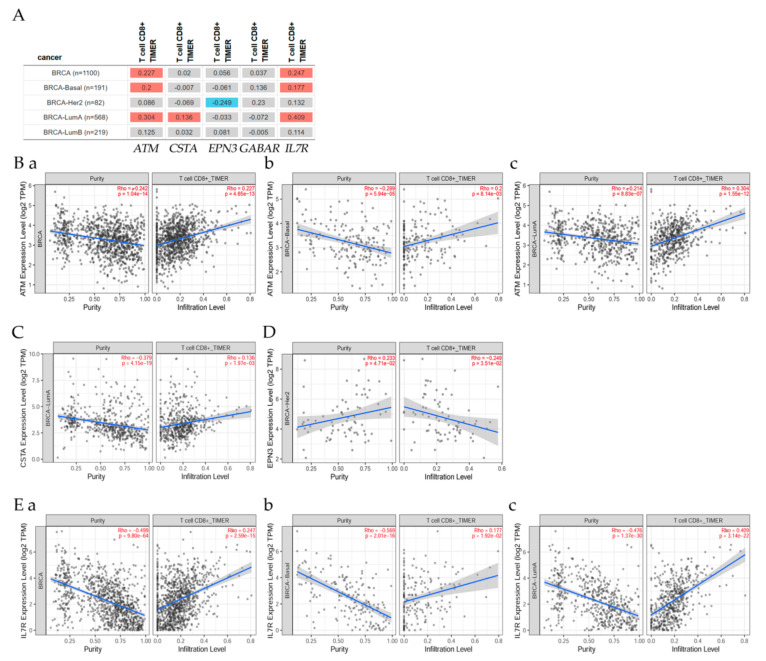
Correlations between gene expression and its immune infiltration levels in diverse breast cancer types. (**A**) The table explores the correlations between gene expression and T cell CD8+ immune infiltrates in BRCA patient types. These figures show the correlation between T cell CD8+ and (**B**) ATM in (**a**) all BRCA, (**b**) BRCA-Basal, and (**c**) BRCA-LumA, (**C**) CSTA in BRCA-LumA, (**D**) EPN3 in BRCA-Her2, (**E**) IL7R in (**a**) all BRCA, (**b**) BRCA-Basal, and (**c**) BRCA-LumA.

**Figure 9:**
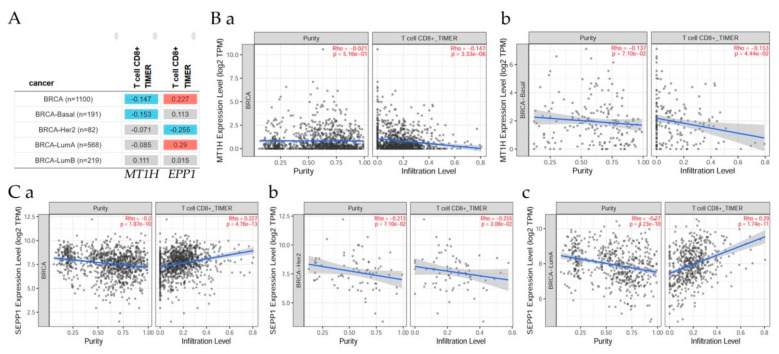
Correlations between gene expression and its immune infiltration levels in diverse breast cancer types. (**A**) The table explores the correlations between gene expression and T cell CD8+ immune infiltrates in BRCA patient types. These figures show the correlation between T cell CD8+ and (**B**) MT1H in (**a**) all BRCA, (**b**) BRCA-Basal, (**C**) SEPP1 in (**a**) all BRCA, (**b**) BRCA-Her2, and (**c**) BRCA-LumA.

The gene module according to data on TIMER2.0 web analysis using Spearman’s *p* values allowed us to select ATM gene and visualize the correlation of its expression with immune infiltration level in diverse breast cancer types as seen in Figure 8 and Figure 9. Results indicated that there was (Figure 8A,B) a positive significant (*p* < 0.05) correlation between ATM gene expression and immune infiltrates, considering the T cell CD8+ by TIMER in all BRCA samples, BRCA-basal and BRCA-LumA; a positive significant (*p* < 0.05) correlation between *stefin A* (Figure 8C) gene expression and immune infiltrates, considering the T cell CD8+ by TIMER in BRCA-LumA; a negative significant (*p* < 0.05) correlation between *epsin 3* (Figure 8D) gene expression and immune infiltrates, considering the T cell CD8+ by TIMER in BRCA-Her2; a non-significant *p* < 0.05) correlation between GABARAB and immune infiltrates, considering the T cell CD8+ by TIMER in all BRCA, BRCA-basal, BRCA-HER, BRCA-LumA; a positive significant (*p* < 0.05) correlation between *IL7R* (Figure 8E(a–c)) gene expression and immune infiltrates, considering the T cell CD8+ by TIMER in all BRCA, BRCA-basal, and BRCA-LumA; a significant (*p* < 0.05) negative correlation between MT1H and immune infiltrates, considering the T cell CD8+ by TIMER in all BRCA, BRCA-basal; a significant (*p* < 0.05) positive correlation between EPP1 and immune infiltrates, considering the T cell CD8+ by TIMER in all BRCA samples, and BRCA-LumA.

The gene module according to data on the web using Spearman’s *p* values allowed us to select the *ATM* gene and visualize the correlation of its expression with immune infiltration level in diverse breast cancer types as seen in Figure 9A,B(a,b),C(a–c). Results indicated that there was a significant (*p* < 0.05) negative correlation between MT1H and immune infiltrates, considering the T cell CD8+ by TIMER in all BRCA, and BRCA-basal patients (Figure 9B(a,b)); a significant positive (*p* < 0.05) correlation between SEPP1 and immune infiltrates, considering the T cell CD8+ by TIMER in all BRCA samples, and BRCA-LumA (Figure 9C(a–c)).

## 8. Conclusions

It can be summarized that in the breast cancer model the cell line transformed only by radiation independently of estrogen was characterized by greater gene expression of *ATM*, *selenoproteins*, *GABA receptor*, *interleukins*, *specifically IL-7*, *epsin*, *stefin* and *metallothioneins* than in other cell lines. Understanding the effect of radiotherapy in different types of cells will help us improve the clinical outcome of radiotherapy. Thus, gene signatures have been demonstrated to be specific to tumor types, hence cell-dependency must be considered in future treatment planning. Molecular and clinical features affect the results of radiotherapy. Thus, using gene technology and molecular information it is possible to improve therapy and reduce the side effects of therapeutic radiation use.

## Data Availability

TIMER2.0 is freely available at http://timer.cistrome.org (accessed on 6 August 2021).
